# Cryopreserved human adipose-derived stromal vascular fraction maintains fracture healing potential via angiogenesis and osteogenesis in an immunodeficient rat model

**DOI:** 10.1186/s13287-021-02182-3

**Published:** 2021-02-04

**Authors:** Tomoyuki Kamenaga, Yuichi Kuroda, Kanto Nagai, Masanori Tsubosaka, Yoshinori Takashima, Kenichi Kikuchi, Masahiro Fujita, Kemmei Ikuta, Kensuke Anjiki, Toshihisa Maeda, Naoki Nakano, Koji Takayama, Shingo Hashimoto, Shinya Hayashi, Takehiko Matsushita, Takahiro Niikura, Ryosuke Kuroda, Tomoyuki Matsumoto

**Affiliations:** Department of Orthopaedic Surgery, Kobe University Graduate School of Medicine, 7-5-1, Kusunoki-chou, 650-0017 Kobe, Japan

**Keywords:** Adipose, Cryopreservation, Fracture, Angiogenesis, Osteogenesis, Rat model

## Abstract

**Background:**

Novel therapeutic strategies for the healing of nonunion, which has serious effects on the quality of life of patients, are needed. We evaluated the therapeutic effect of local transplantation of human stromal vascular fraction (SVF) cells on fracture healing in a rat non-healing fracture model and compared the effects between freshly isolated (F) and cryopreserved (C)-SVFs.

**Methods:**

Non-healing fracture model was induced in the femur of female immunodeficient rats (F344/N Jcl rnu/rnu) with cauterizing periosteum. Immediately after the creation of non-healing fracture, rats received local transplantation of F and C-SVFs suspended in phosphate-buffered saline (PBS) or the same volume of PBS without cells using the same scaffold as a control group. During 8 weeks post-surgery, radiologic, histological, immunohistochemical, and biomechanical analyses were performed to evaluate fracture healing. The comparison of radiological results was performed with a chi-square test, and the multiple comparisons of immunohistochemical, histological, and biomechanical results among groups were made using a one-way analysis of variance. A probability value of 0.05 was considered to denote statistical significance.

**Results:**

At week 8, in 60% of animals receiving F-SVF cells and in 50% of animals receiving C-SVF cells, the fracture radiologically healed with bone union whereas nonunion was observed in the control group. The healing potential was also confirmed by histological and biomechanical assessments. One of the mechanisms underlying healing involving intrinsic angiogenesis/osteogenesis was enhanced in F- and C-SVF groups compared with that in the control group. Human cell-derived vasculogenesis/osteogenesis, which was also confirmed in an in vitro differentiation assay, was also enhanced in the F- and C-SVF groups compared with that in the control groups and could be another mechanism for healing.

**Conclusions:**

SVF cells can enhance bone healing and cryopreserved cells have almost equal potential as fresh cells. SVF cells can be used for improving nonunion bone fracture healing as an alternative to other mesenchymal stem cells and the effect of SVF cells can be maintained under cryopreservation.

## Background

In fracture repair, 5–10% of closed fractures and 17% of open long bone fractures result in nonunion, which severely diminishes the quality of life of patients [1–3]. Therefore, the establishment of novel therapeutic strategies to promote the healing of nonunion fractures is warranted. A sufficient blood supply to the fracture site, neovascularization, and osteogenesis are essential for the formation of new bone and avoiding nonunion of a fracture [4–7]. Recently, stem cell-based therapy has become a useful option to promote fracture healing [8, 9]. Evidence has shown that transplantation of bone marrow-derived stromal cells promote the healing of fractures via angiogenesis and osteogenesis [10–12]. However, such autologous cell therapies require the collection of a large number of cells from the patient and the use of a rather invasive procedure [10].

Adipose tissue has increasingly been garnering attention as a promising source of undifferentiated mesenchymal stem cells [13]. Adipose-derived stem cells (ADSCs) have multilineage potential equivalent to that of bone marrow-derived stem cells and can be easily obtained in large amounts from subcutaneous adipose tissue [14, 15]. However, the preparation of ADSCs involves cell culture and the process takes a few weeks from cell isolation to therapeutic application. Cells isolated from the stromal vascular fraction (SVF) of enzymatically digested adipose tissue, which are referred to as adipose-derived regenerative cells, include ADSCs, macrophages, pericytes, fibroblasts, blood cells, and vessel-forming cells, such as endothelial and smooth muscle cells and their progenitors [16–18]. Preparation of SVF cells for transplantation, which can be available within 3–4 h after tissue collection, involves cell separation, seeding of scaffold cells, and a one-stage surgical treatment at the same time as fracture repair surgery [19]. This reduction in the time required for the overall process can facilitate the practical use of SVF cells and bypass biological and regulatory issues associated with extensive ex vivo processing and cellular expansion. The efficacy of ADSCs or SVF therapy has been reported in several clinical fields, including cardiology, urology, and plastic and reconstructive surgery [20–23]. In orthopedics, the effects of SVF therapy for knee osteoarthritis have been well described [24–29]. However, there is scant evidence of bone healing with the use of SVF cells [30], thus the applicability of SVF cells for the healing of fractures remains to be explored.

As clinical therapeutic applications of SVF cells continue to expand, the rapid development of cell banking is expected in future clinical scenarios. Cryopreservation is an appropriate solution since SVF cells can be easily frozen and stored, while maintaining the proliferative capacity and differentiation potential [31]. Recent studies have demonstrated the therapeutic potential of rat SVF for bone fracture in 8 human patients [32]; while cryopreserved SVF cells have been shown to facilitate bone healing in an equine carpal chip fracture [33] and rat bone defect model [34]. However, no study has compared the therapeutic benefits of fracture healing using the same animal model between freshly isolated and cryopreserved SVFs.

In this study, we evaluated the therapeutic effects of transplantation of human SVF cells for fracture healing in a rat model of non-healing fracture and compared the therapeutic effects of freshly isolated (F) and cryopreserved (C)-SVF cells.

## Methods

### Preparation of SVF cells

Human SVF cells were extracted from 10 female donors (mean age, 65.2 ± 5.5 years; body mass index, 25.5 ± 3.0 kg/m^2^) undergoing intra-articular injection of SVF for treatment of knee osteoarthritis using the Celution® 800/CRS system (Cytori Therapeutics Inc., San Diego, CA, USA). The collection of human SVF cells was approved by the local Institutional Review Board and informed consent was obtained from all donors. All subjects underwent a liposuction procedure under general anesthesia, and 100–360 mL of adipose tissue was obtained. The extracted tissue was then processed using the Celution® 800/CRS System in accordance with a previously described method [29]. Briefly, subcutaneous adipose tissue was removed, minced, and then digested with a mixture of highly purified collagenase. After digestion, SVF cells were concentrated by centrifugation at 1500 rpm for 5 min, extracted from the system, and counted. Thereafter, the cells were suspended in 100 μL of phosphate-buffered saline (PBS) for fresh use. The remaining cells were cryopreserved for 3 months before local transplantation as described previously [14]. Briefly, SVF cells were isolated and frozen in 20% human serum albumin (10 g/50 mL, Nihon Pharmaceutical Co., Ltd., Tokyo, Japan) and 10% dimethyl sulfoxide (DMSO) in lactated Ringer’s solution at − 80 °C and then cooled at − 1 °C/min from 4 °C to − 50 °C, and at − 10 °C/min to − 80 °C. Prior to transplantation, the SVF cells were thawed in a water bath at 37 °C for 2 min, washed rapidly, and suspended in 10 volumes of PBS. Afterward, the cells were centrifuged at 1500 rpm for 6 min, washed in PBS, and suspended in 100 μL of PBS.

### Cell viability

Cell viability was calculated using the NC-100™ NucleoCounter® Automated Cell Counting System (ChemoMetec A/S, Allerod, Denmark). The total cell count and the count of non-viable cells were determined by staining of cell nuclei with propidium iodide before and after lysis of the cell membrane [29].

### Operative procedures

**Fig. 1:**
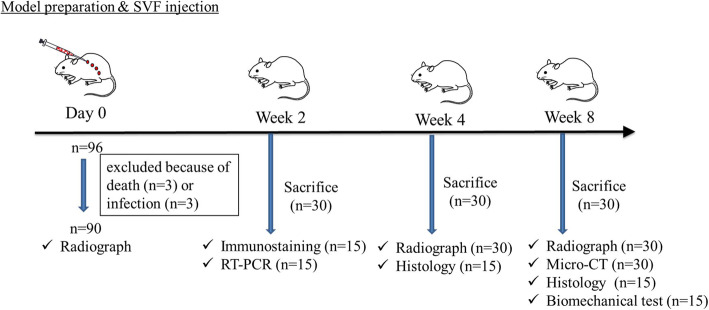
A schematic of the study design, the time course of the study, and the number of animals at each stage. SVF, stromal vascular fractions

Female athymic nude rats (F344/N Jcl rnu/rnu; age, 9 weeks; body weight, 140–160 g) were obtained from CLEA Japan (Tokyo, Japan). The protocols for all animal procedures were approved by the local Ethics Committee (Permission No; P150701) and conducted in accordance with the Japanese Physiological Society Guidelines for the Care and Use of Laboratory Animals. Anesthesia was induced by intra-peritoneal administration of a mixture of ketamine hydrochloride (60 mg/kg) and xylazine hydrochloride (10 mg/kg). Non-healing femoral fractures were induced by cauterizing the periosteum around the fracture site [35, 36]. Immediately after fracture induction, the rats received local transplantation of 1.0 × 10^5^ human F- or C-SVFs suspended in 100 μL of PBS using atelocollagen gel (Koken Co., Ltd., Tokyo, Japan), or the same volume of PBS without cells using the same scaffold as a control group (*n* = 10/group). The rats were euthanized with an overdose of ketamine and xylazine for biomechanical and histological analyses, and the femurs were directly frozen for biomechanical analysis or snap frozen in liquid nitrogen and stored at − 80 °C for histological analysis over the indicated time course (Fig. 1). Six rats were excluded due to death or infection. All three dead animals were included in the initial 20 cases of the operation and died on the day of surgery or the day after surgery. All three infections occurred within 2 weeks after surgery. Finally, remained 90 rats were included for analysis.

### Radiologic assessment

Radiographs of the fractured legs of anesthetized rats fixed in the supine position were obtained at postoperative weeks (POW) 0, 4, and 8 (*n* = 10/group). Fracture union was identified by the presence of bridging callus on two cortices [37]. The radiographs of each animal were examined by two observers (YT and MF) blinded to the course of treatment. To evaluate the fracture healing process, relative callus areas around the fracture sites on radiographs at each time point were quantified using ImageJ software (National Institutes of Health, Bethesda, MD, USA).

### Micro-computed tomography (CT) assessment

To quantify callus formation, micro-CT of the harvested fractured legs of five rats was performed for each group at POW 8 using a micro-CT imager (R_mCT2 FX; Rigaku Corporation, Tokyo, Japan), as described previously [38]. Three-dimensional reconstruction of the radiographs was performed using built-in software. The region of interest was defined as an area extending 3 mm proximally and distally to the fracture line. The following parameters of the callus were calculated from the region of interest using bone microstructure software (TRI/3D-BON-FCS64; Ratoc System Engineering, Tokyo, Japan): total tissue volume, callus bone mineral content, and bone volume fraction (the ratio of bone volume to tissue volume). Callus bone mineral content was calibrated by scanning hydroxyapatite phantoms of known densities that were provided by the system manufacturer.

### Histological assessment

Samples were sectioned to thicknesses of 6 mm, mounted on slides, and fixed with 4% paraformaldehyde at 4 °C for 5 min. Hematoxylin and eosin (HE) and safranin-O staining were performed to histologically evaluate the endochondral ossification of five animals in each group on POW 4 and 8. The degree of fracture healing was evaluated using the 5-point scale proposed by Allen et al. [39].

### Biomechanical analysis of fracture union

Biomechanical evaluation of five rats from three groups was performed at POW 8. Fractured femurs and the contralateral non-fractured femurs were harvested. After removal of the intra-medullary fixation pins, a standardized 3-point bending test was performed using a load torsion and bending tester (MZ-500S; Maruto Instrument Co., Ltd., Tokyo, Japan) as previously described [40]. Each bone was positioned with the posterior surface downward and force was applied directly to the fracture site with crosshead at a speed of 2 mm/min until rupture occurred. The load and displacement were analyzed and recorded using an attached computer and software supplied with the testing machine during the 3-point bending test. The ultimate stress (N), extrinsic stiffness (N/mm), and failure energy (Nmm) were calculated using a load-deformation curve. The percentage ratio of each parameter of the fractured (right) femur vs. the unfractured (left) femur was calculated for each rat from the load deflection curve. The relative ratio of the fractured (right) femur to the non-fractured (left) femur was calculated for each group and averaged.

### Assessment of intrinsic angiogenesis and osteogenesis

Fluorescent immunostaining of the rat endothelial cell (EC) marker isolectin B4 was performed at POW 2 to quantify the regenerated capillaries and to evaluate neovascularization (*n* = 5/group). Osteogenesis was assessed with antibodies against the rat osteocalcin (OC) antigen (200 mg/mL, Santa Cruz Biotechnology, Inc., Dallas, TX, USA, SC18319) to identify rat osteoblasts (OBs) at POW 2 (*n* = 5/group), as previously described [41]. Briefly, rat OBs were incubated with fluorescein isothiocyanate (FITC)-conjugated primary antibodies against isolectin B4 (Vector Laboratories, Burlingame, CA, USA, FL1201) and the rat OC (dilution, 1:100) at room temperature for 1 h followed by Alexa Fluor 488-conjugated donkey anti-goat IgG antibody (dilution, 1:200; Life Technologies, Carlsbad, CA, USA, A11055) at room temperature for 2 h. Nuclei were counter-stained with 4′,6-diamidino-2-phenylindole (DAPI) solution (dilution, 1:100; Dojindo Laboratories Co., Ltd., Kumamoto, Japan) for 5 min. The number of capillaries was determined by staining of isolectin B4 and OC-positive cells in five randomly selected fields in each section and counted under a fluorescent microscope, and the counts were averaged.

### Assessment of human cell-derived vasculogenesis and osteogenesis

Following transplantation of human cells, double immunofluorescence staining (*n* = 5/group) was performed to evaluate vasculogenesis and osteogenesis at the fracture site at POW 2 and 8 using anti-human CD31 (hCD31) anti-mouse antibody (dilution, 1:100; Santa Cruz Biotechnology, SC53411), anti-human OC (hOC) anti-rabbit antibody (dilution, 1:100; Santa Cruz Biotechnology, SC30044), and anti-human nuclear antigen (hNA) anti-mouse antibody (dilution, 1:100; EMD Millipore Corporation, Billerica, MA, USA, MAB1281). In addition, rat-specific isolectin B4-FITC-conjugated antibody was used to detect the existence of rat ECs. The samples were incubated with the primary antibodies at room temperature for 1 h and then with Alexa Fluor 594-conjugated goat anti-mouse (R37121) or Alexa Fluor 488-conjugated goat anti-rabbit IgG (1200; Life Technologies, A11008) at room temperature for 2 h to detect hCD31 and hNA or hOC, respectively. Finally, the nuclei were counter-stained with a DAPI solution (dilution, l:100) for 5 min. Cells positive for hNA and hOC at the fracture site were accepted as differentiated human OBs. The numbers of rat ECs positive for isolectin B4 and human-derived differentiated ECs positive for hCD31 were compared, as were the numbers of rat OBs and differentiated human OBs.

To verify no antibodies species cross-reactivity, negative primary antibody controls, using PBS rather than the primary antibodies, in the dual labeling were conducted.

### Assessment of gene expression

Real-time polymerase chain reaction (RT-PCR) was performed to assess the expression levels of rat-specific marker genes (BMP-2, HIF1-a, and VEGF) at POW 2 in five rats from each group. Granulated and callus tissues surrounding the fracture sites were harvested at POW 2. Total RNA was extracted from tissue using the RNeasy Mini Kit (Qiagen, Valencia, CA, USA) and reverse-transcribed into cDNA using the High-Capacity cDNA Reverse Transcription Kit (Applied Biosystems, Foster City, CA, USA). RT-PCR amplification of the cDNA was performed in triplicate using SYBR Green reagent (Applied Biosystems) and an ABI PRISM 7700 Sequence Detection System (Thermo Fisher Scientific, Waltham, MA, USA). Relative gene expression was normalized against the housekeeping gene glyceraldehyde 3-phosphate dehydrogenase using the comparative cycle threshold method [42].

### In vitro assessment of the differentiation potential of ECs

The differentiation potential of ECs was assessed as described previously [43]. Briefly, ECs were cultured in the wells of 12-well plates at a density of 1.0 × 10^4^ cells/well in endothelial growth medium supplied with the EGM™-2 Endothelial Cell Growth Medium BulletKit™ (Lonza Biologics, Portsmouth, NH, USA) with four replicates (EC basic medium, hydrocortisone, fibroblast growth factor-basic, vascular endothelial growth factor, recombinant human long R3 insulin-like growth factor-1, ascorbic acid, epidermal growth factor, gentamicin, amphotericin-B [GA]-1000, and heparin) supplemented with 10% fetal bovine serum (FBS) and then incubated at 37 °C under an atmosphere of 5% CO_2_/95% air for 1 week. To demonstrate the ability of ECs to take up 1,1′-dioctadecyl-3,3,3′,3′-tetramethylindocarbocyanine (DiI)-labeled acetylated low-density lipoproteins (acLDLs) (Biomedical Technologies, Inc., Stoughton, MA, USA) and to bind to lectin extracted from *Ulex europaeus* (Molecular Probes, Eugene, OR, USA), the ECs were first incubated with DiIacLDLs (10 mg/mL) at 37 °C for 4 h and then fixed with 1% paraformaldehyde for 10 min. After washing, the ECs were continuously incubated with FITC-labeled lectin extracted from *U. europaeus* (10 mg/mL) for 1 h, then mounted using DAPI mounting medium, and viewed under an inverted fluorescence microscope. ECs positively stained for uptake of acLDLs and binding of lectin extracted from *U. europaeus* were counted in five randomly selected fields in each section, and the counts were averaged. The formation of endothelial tubular structures was also assessed in vitro using Matrigel cell culture matrix (BD Biosciences, San Jose, CA, USA). Briefly, ECs cultured in endothelial basal medium-2 were seeded into the wells of 48-well plates coated with Matrigel cell culture matrix and cultured at 37 °C for 48 h. Following, tubular formation was observed under a microscope and the total tube length was calculated from three randomly selected low-power fields of each plate.

### In vitro assessment of osteogenic differentiation potential

Osteogenic differentiation assays were performed as previously reported [15, 43, 44]. Briefly, monolayer cultures of F- and C-SVF cells were cultured in α-minimum essential medium (Invitrogen Corporation, Carlsbad, CA, USA) supplemented with 10% FBS, 100 U/mL of penicillin/streptomycin solution, 0.1 mM dexamethasone, 50 mM ascorbate-2-phosphate, and 10 mM β-glycerophosphate (all, Sigma-Aldrich Corporation, St. Louis, MO, USA) and incubated at 37 °C under an atmosphere of 5% CO2/95% air. To assess the capability to undergo osteogenesis, the cells were cultured in osteogenic medium at a density of 1.0 × 10^5^ cells/well in four replicates. The medium was changed every 3 days. Osteogenesis was assessed by staining for alkaline phosphatase (ALP) on day 14 and alizarin red staining on day 21. On day 14, ALP was collected from the cells in a monolayer culture with the osteogenic medium. After centrifugation at 1500 rpm for 5 min, the supernatant was collected for measurement of ALP [45]. To quantify the induced mineralization in alizarin red staining, the bound stain was dissolved in cetylpyridinium chloride monohydrate (Sigma-Aldrich Corporation, St. Louis, MO, USA) and measured at an optical density of 550 nm [46].

### Statistical analysis

All values are reported as the mean ± standard deviation (SD). All analyses were conducted using StatView 5.0 software (Abacus Concepts, Inc., Berkeley, CA, USA). Comparisons among three groups were performed using the chi-squared test or one-way analysis of variance followed by post hoc Tukey’s test. Comparisons between two groups were made using Mann–Whitney *U* test. A probability (*p*) value of < 0.05 was considered statistically significant.

## Results

### Cell viability

In total, 5.1 × 10^7^ ± 2.0 × 10^7 SVF^ cells were obtained by liposuction and purified. There was no significant difference in the ratio of viable SVF cells between the F- and C-SVF groups (89.6% ± 2.8% vs. 83.6% ± 2.2%, respectively, *p* = 0.29).

### Radiologic and micro-CT assessment

**Fig. 2:**
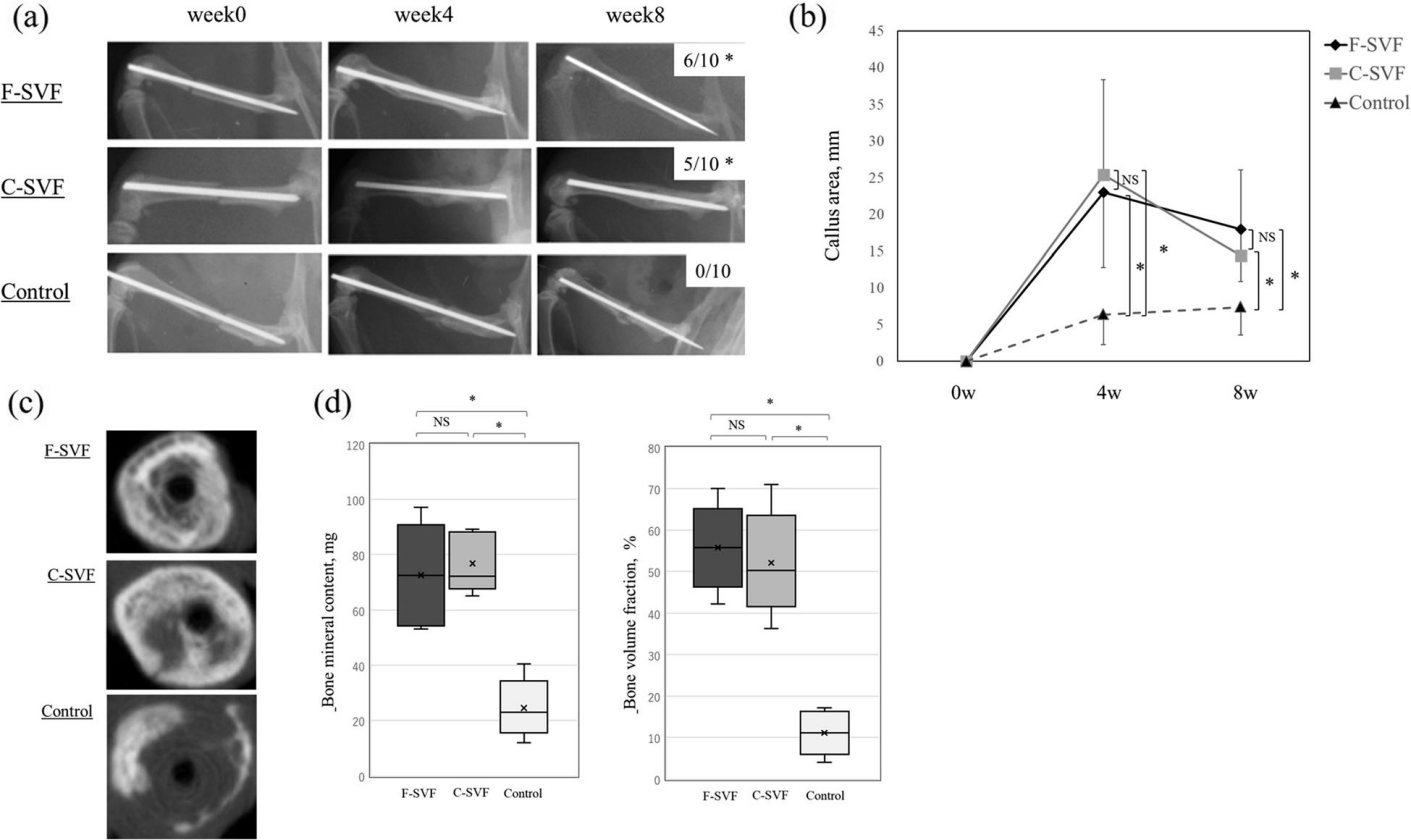
**a** Representative plain radiographs for each group (*n* = 10/group). **b** Comparison of the callus areas of the three groups (*n* = 10/group). Data are presented as the mean ± SD. **c** Representative micro-CT axial images of the three groups. **d** Quantitative comparison of bone mineral content and volume fraction among the three groups (*n* = 10/group). *; *p* < 0.05 for F- or C-SVF vs. control; NS, not significant. F-SVF, freshly isolated stromal vascular fraction; C-SVF, cryopreserved stromal vascular fraction

Representative radiographs of the fractured sites and the fracture healing ratio of each group are shown in Fig. 2a. At POW 8, the fracture was radiologically healed with formation of bridging callus and bone union in 60% (6/10) of rats receiving F-SVF and 50.0% (5/10) receiving C-SVF. There was no significant difference in the ratio of bone union between the two groups, whereas the fracture sites of all rats in the control group failed to unite and showed no formation of bridging callus. The frequency of morphological fracture healing in the control group was significantly lower than in the F- and C-SVF groups. Moreover, the callus area was significantly larger in both the F- and C-SVF groups than in the control group at POW 4 and 8, whereas there was no significant difference between the F- and C-SVF groups (Fig. 2b) (POW 4: F-SVF, 23.0 ± 0.2 mm^2^; C-SVF, 25. 4 ± 12.9; control, 6.4 ± 4.1, respectively; not significant for F-SVF vs. C-SVF; *p* < 0.05 for the F- or C-SVF group vs. the PBS [control] group; POW 8: F-SVF, 18.0 ± 7.1; C-SVF, 14.4 ± 11.4; control, 7.4 ± 3.8, respectively; not significant for F-SVF vs. C-SVF; *p* < 0.05 for F- or C-SVF vs. control). Bone union was confirmed by the disappearance of the fracture line in the F- and C-SVF groups by micro-CT, as shown in representative axial views of the fracture sites presented in Fig. 2c. The bone mineral content and bone mineral density were significantly higher in both SVF groups than in the control group at POW 8, while there was no significant difference in either parameter between the F- and C-SVF groups (Fig. 2d; bone mineral content (mg): F-SVF, 72.5 ± 21.1; C-SVF, 76.7 ± 18.8; control, 24.5 ± 10.5, respectively; not significant for F-SVF vs. C-SVF; *p* < 0.05 for F- or C-SVF vs. control; bone volume fraction (%): F-SVF, 55.7 ± 10.2; C-SVF, 52.2 ± 12.5; control, 11.1 ± 5.5, respectively; not significant for F-SVF vs. C-SVF; *p* < 0.05 for F- or C-SVF vs. control).

### Histological assessment

**Fig. 3:**
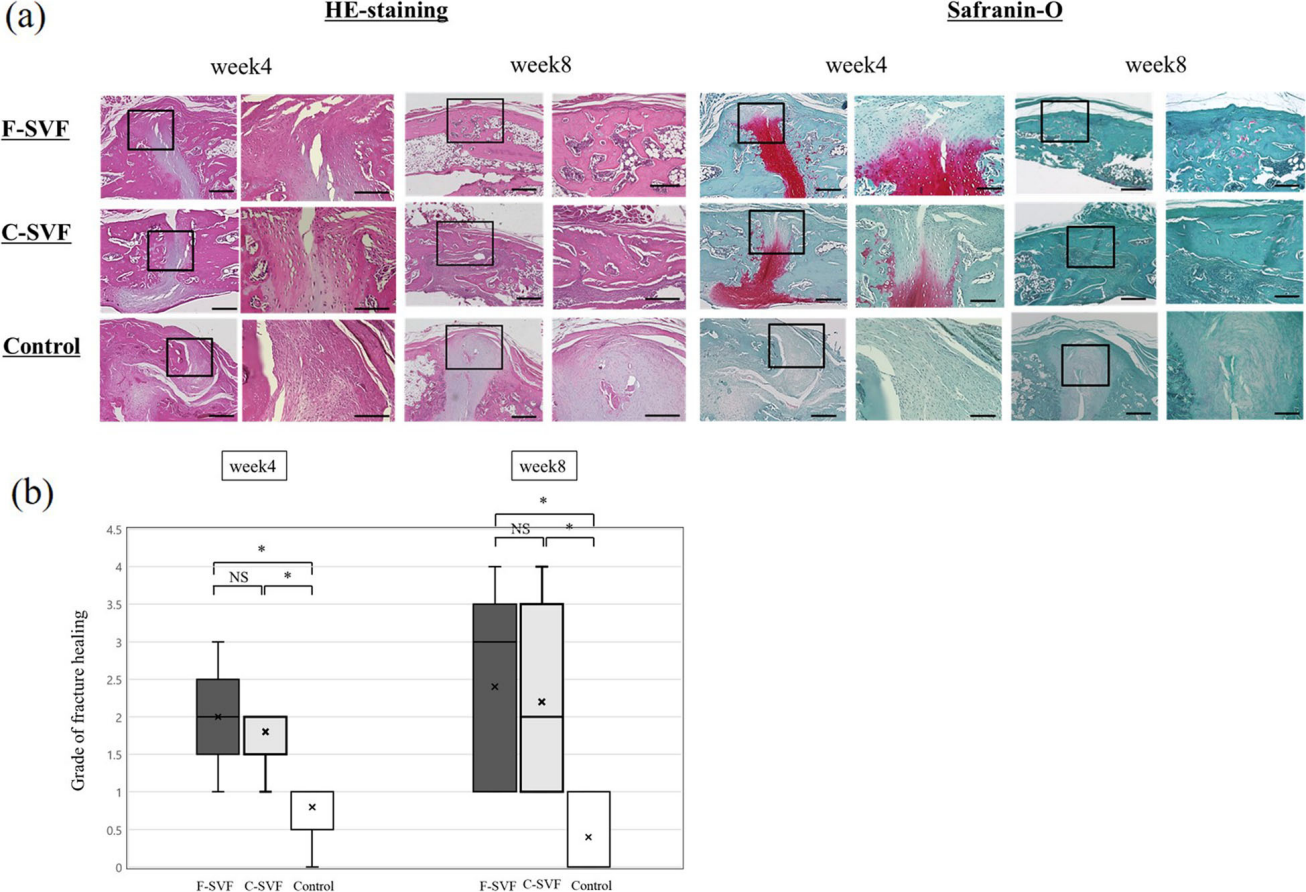
**a** Representative histologic sections of the fracture sites stained with HE and safranin-O/fast green of the three groups. **b** Comparison of the degree of fracture healing using a 5-point scale among the three groups (*n* = 5/group). *; *p* < 0.05 for F- or C-SVF vs. control; NS, not significant. F-SVF, freshly isolated stromal vascular fraction; C-SVF, cryopreserved stromal vascular fraction

Histological evaluations with HE and safranin-O staining demonstrated complete union at POW 8 after the formation of bridging cartilage callus at POW 4 in the F- and C-SVF groups. In contrast, although the formation of a thick callus was observed, the callus was finally absorbed, but the fracture gaps were not filled with the bridging callus and the granulation tissues were not joined at POW 8 in the control group (Fig. 3a). There was no significant difference in the degree of fracture healing between the F- and C-SVF groups at POW 4 and 8, as assessed in accordance with the classification scheme proposed by Allen et al. [39]. However, the extent of fracture healing was significantly greater in the F- and C-SVF groups as compared to the control group at POW 4 and 8 (Fig. 3b) (POW 4: F-SVF, 2.0 ± 0.71; C-SVF, 1.8 ± 0.45; control, 0.8 ± 0.45, respectively; not significant for F-SVF vs. C-SVF group; *p* < 0.05 for F-SVF vs. control and C-SVF vs. control; POW 8: F-SVF, 2.4 ± 1.3; C-SVF, 2.2 ± 1.3; control, 0.8 ± 0.45, respectively; not significant for F-SVF vs. C-SVF; *p* < 0.05 for F-SVF vs. control and C-SVF vs. control).

### Biomechanical assessment of fracture healing

**Fig. 4:**
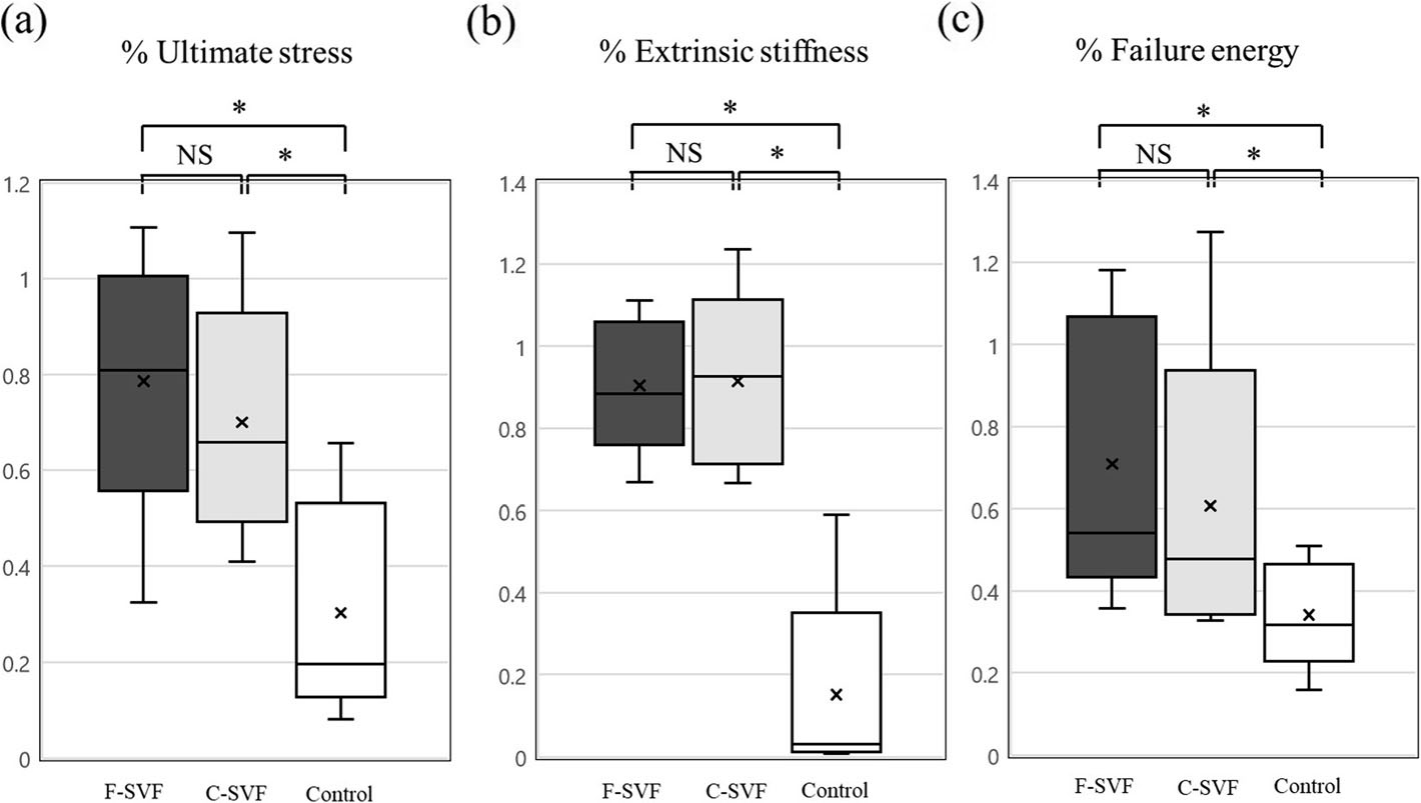
Results of functional recovery after fracture assessment by the biomechanical three-point bending test at POW 8 (*n* = 5/group). Comparison of the percentage of each parameter (**a** ultimate stress; **b** failure energy; **c** extrinsic stiffness) indicating the ratio of each value at the fracture sites to contralateral intact sites. *; *p* < 0.05 for F- or C-SVF vs. control; NS, not significant. F-SVF, freshly isolated stromal vascular fraction; C-SVF, cryopreserved stromal vascular fraction

The ultimate stress, extrinsic stiffness, and failure energy values of the fractured femur vs. the contralateral intact femur were significantly higher in the F- and C-SVF groups than the control group, while there were no significant differences between the F- and C-SVF groups (Fig. 4a–c; ultimate stress: F-SVF, 0.79 ± 0.29; C-SVF, 0.71 ± 0.26; control, 0.29 ± 0.21, respectively; not significant for F-SVF vs. C-SVF; *p* < 0.05 for F-SVF vs. control and C-SVF vs. control; extrinsic stiffness: F-SVF, 0.88 ± 0.25; C-SVF, 0.94 ± 0.36; control, 0.15 ± 0.14, respectively; not significant for F-SVF vs. C-SVF; *p* < 0.05 for F-SVF vs. control and C-SVF vs. control; failure energy: F-SVF, 0.71 ± 0.34; C-SVF, 0.65 ± 0.36; control, 0.34 ± 0.13, respectively; not significant for F-SVF vs. C-SVF; *p* < 0.05 for F-SVF vs. control and C-SVF vs. control).

### Intrinsic vascularization and osteogenesis at POW 2

**Fig. 5:**
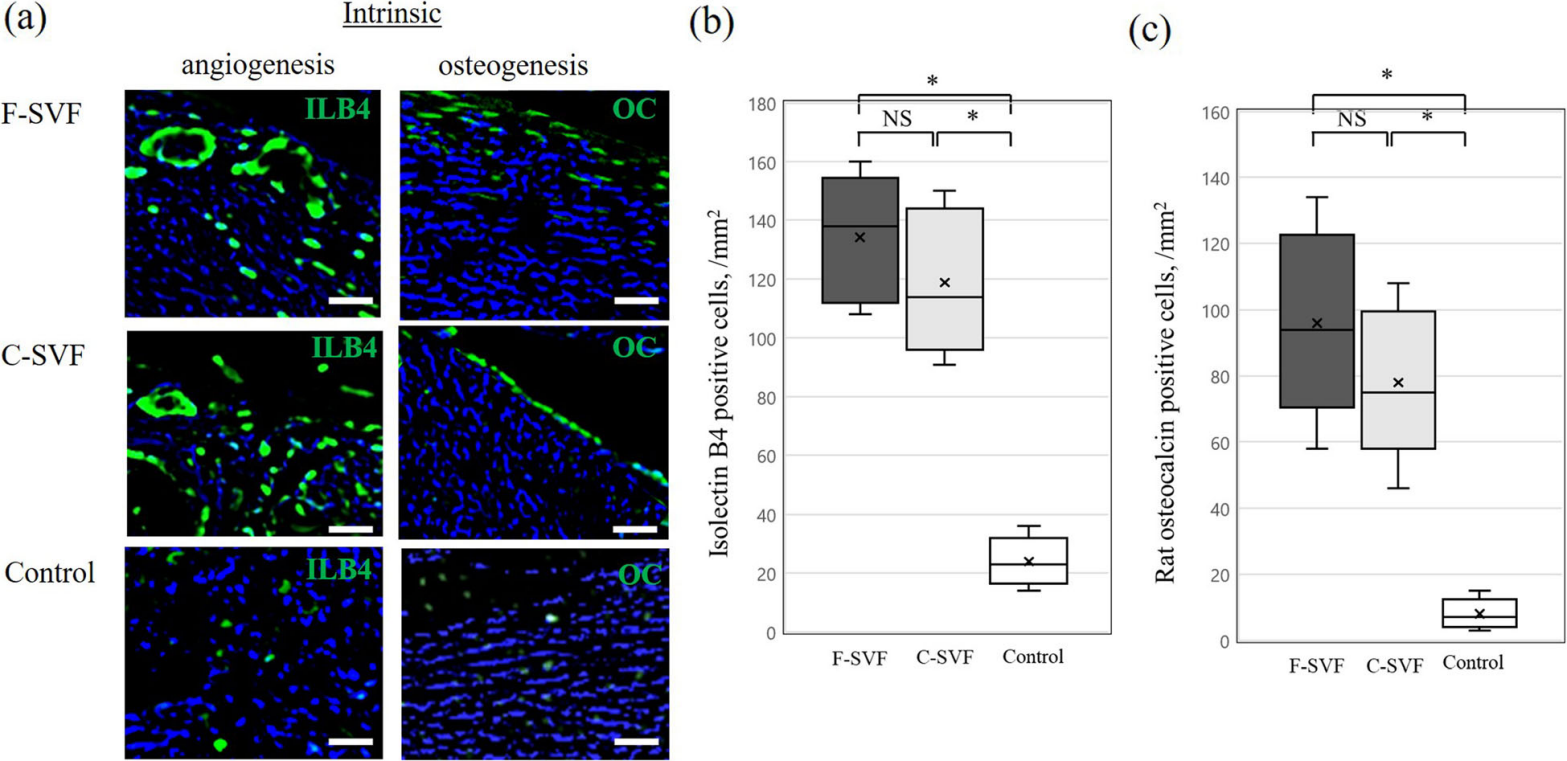
**a** Representative intrinsic vascular staining of isolectin B4 (ILB4; green) and osteoblast staining with rat osteocalcin (OC; green). Scale bar: 50 mm. **b** Quantitative analysis of neovascularization assessed by the capillary density of the three groups (*n* = 5/group). **c** Quantitative analysis of osteogenesis assessed by osteoblast density of the three groups (*n* = 5/group). *; *p* < 0.05 for F- or C-SVF vs. control; NS, not significant. F-SVF, freshly isolated stromal vascular fraction; C-SVF, cryopreserved stromal vascular fraction

Vascular staining with isolectin B4 showed a marked increase in ECs at the fracture site in the F- and C-SVF groups as compared to the control group at POW2 (Fig. 5a). The capillary density assessed by isolectin B4-positive cells was significantly greater in the F- and C-SVF groups than in the control group (F-SVF: 134.0 ± 21.8/mm^2^; C-SVF: 118.0 ± 24.4/mm^2^; control: 24.0 ± 8.4/mm^2^; not significant for F-SVF vs. C-SVF; *p* < 0.05 for control vs. F-SVF or C-SVF; Fig. 5b). At the fracture site, the ratio of OB-positive cells was significantly greater in the F- and C-SVF groups as compared with the control group, with enhanced intrinsic osteogenesis observed lining the new bone surface (F-SVF: 96.0 ± 28.6/mm^2^; C-SVF; 78.0 ± 22.8/mm^2^; control: 8.0 ± 4.8/mm^2^; not significant for F-SVF vs. C-SVF; *p* < 0.05 for control vs. F-SVF or C-SVF; Fig. 5c).

### Human cell-derived vasculogenesis and osteogenesis

**Fig. 6:**
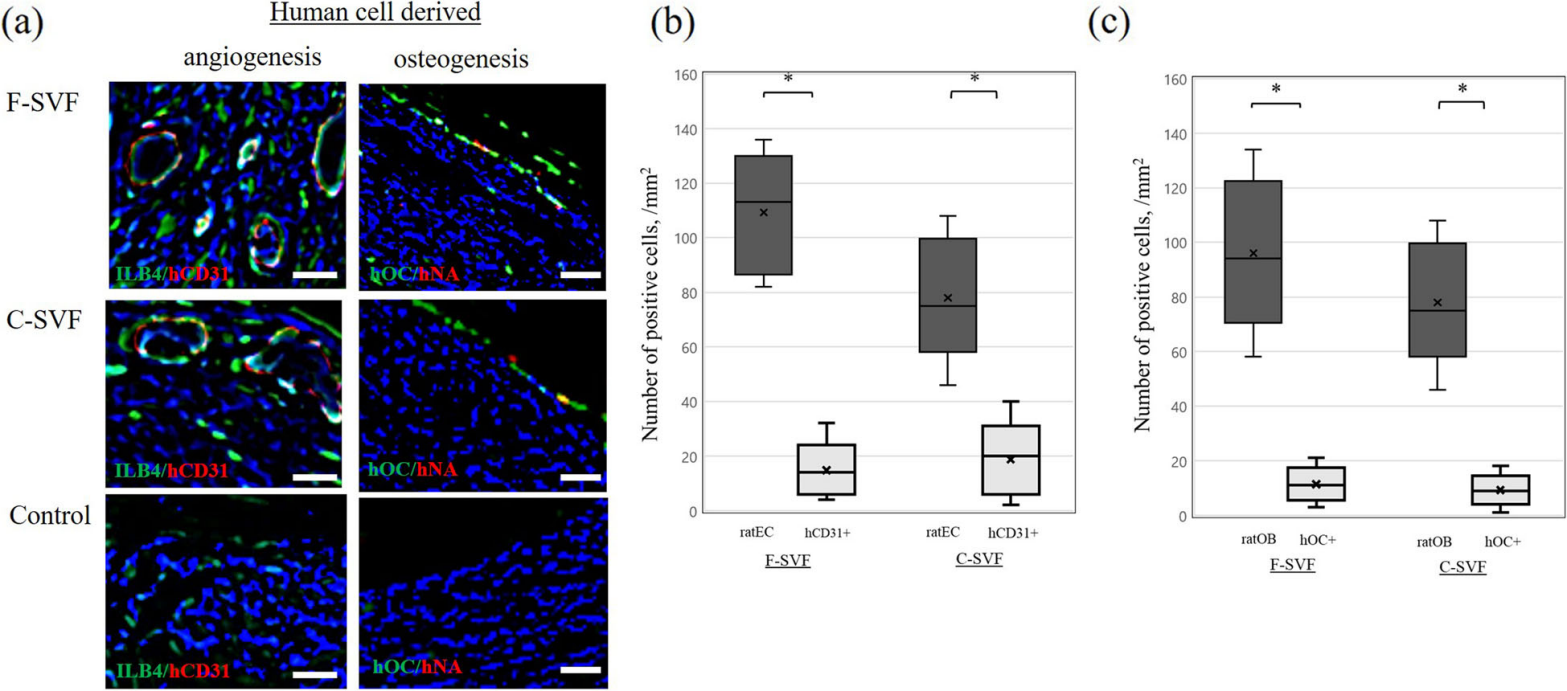
**a** Representative double immunostaining for human-CD31 (hCD31; red) and rat-isolectin B4 (ILB4; green), and immunostaining for hNA (red) and hOC (green) in the three groups. Scale bar: 50 mm. **b** Comparison of the number of isolectin B4-postitive rat ECs and differentiated hCD31-positive human-derived ECs in the F- and C-SVF groups (*n* = 5/group). **c** Comparison of the number of rat osteoblasts (rat OBs) and differentiated human OBs in the F- and C-SVF groups (*n* = 5/group). *; *p* < 0.05 for F- or C-SVF vs. control; NS, not significant. F-SVF, freshly isolated stromal vascular fraction; C-SVF, cryopreserved stromal vascular fraction

Immunostaining of human ECs at POW 2 revealed differentiated hCD31 and ECs at the fracture sites in the F- and C-SVF groups (Fig. 6a). Moreover, differentiated hNA and hOC double-positive OBs were markedly more prevalent in the newly formed bone surface in the F- and C-SVF groups than the control group. However, there were significantly fewer differentiated hCD31-positive ECs than isolectin B4-positive rat ECs in the F- and C-SVF groups (Fig. 6b), and significantly fewer differentiated human OBs than OC-positive rat OBs in the F- and C-SVF groups (Fig. 6c). No differentiated human ECs and OBs were observed at POW 8. Negative control experiments showed minimal background staining attributed to the secondary antibodies.

### Assessment of gene expression

**Fig. 7:**
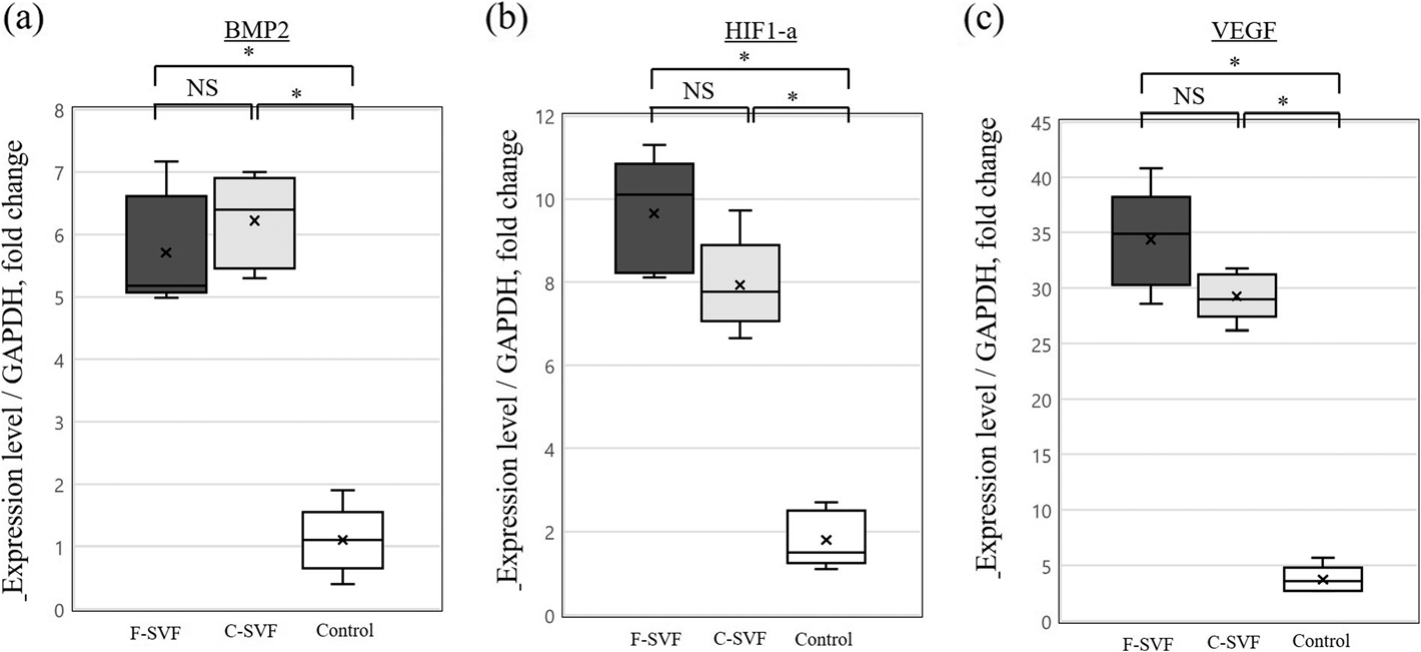
**a** Expression of BMP-2, **b** HIF1-a, and **c** VEGF in each group at POW 2, as determined by RT-PCR (*n* = 5/group). *; *p* < 0.05 for F- or C-SVF vs. control; NS, not significant. F-SVF, freshly isolated stromal vascular fraction; C-SVF, cryopreserved stromal vascular fraction

RT-PCR revealed that the expressions levels of BMP-2, HIF1-a, and VEGF at POW 2 were greater in the F- and C-SVF groups than the control group, while there were no significant differences between the F- and C-SVF groups (Fig. 7a–c; BMP-2: F-SVF, 5.70 ± 0.91-fold; C-SVF, 6.2 ± 0.75; control, 1.1 ± 0.55, respectively; not significant for F-SVF vs. C-SVF; *p* < 0.05 for F-SVF vs. control and C-SVF vs. control; HIF1-a: F-SVF, 9.65 ± 1.45-fold; C-SVF, 7.92 ± 1.12; control, 1.80 ± 0.67, respectively; not significant for F-SVF vs. C-SVF; *p* < 0.05 for F-SVF vs. control and C-SVF vs. control; VEGF: F-SVF, 34.43 ± 4.45-fold; C-SVF, 29.24 ± 2.28; control, 3.75 ± 1.20, respectively; not significant for F-SVF vs. C-SVF; *p* < 0.05 for F-SVF vs. control and C-SVF vs. control).

### In vitro differentiation potential of SVF cells

**Fig. 8:**
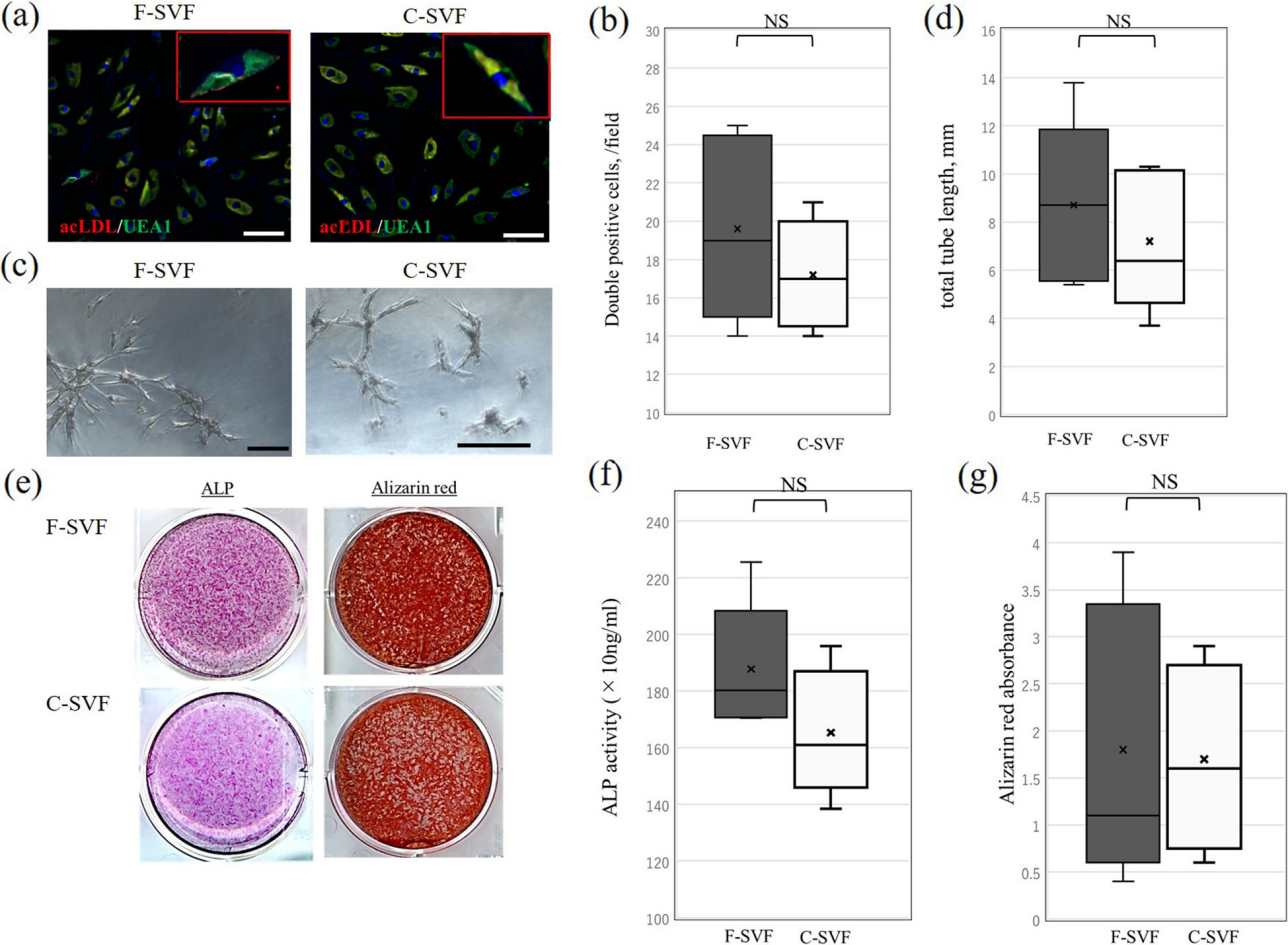
Comparison of the angiogenic and osteogenic differentiation potential of F- and C-SVFs (n = 5/group). Double-positive staining for uptake of acLDLs and binding of lectin extracted from *U. europaeus* was observed in the F- and C-SVF groups (**a**) (bar, 50 mm), without significant difference between the two groups (**b**). Based on the assessment of the tube length, both the F- and C-SVF groups had high potential (**c**) (bar, 100 mm) with no significant difference between the groups (n = 5/group) (**d**). Both groups were positive for ALP and alizarin red staining (**e**) with no significant differences of ALP activity (**f**) and alizarin red mineralization (**g**) between the F- and C-SVF groups (n = 5/group). NS, not significant. F-SVF, freshly isolated stromal vascular fraction; C-SVF, cryopreserved stromal vascular fraction

Double-positive staining for the uptake of acLDLs and binding of lectin extracted from *U. europaeus* were observed in both the F- and C-SVF groups, without significant differences between the F- and C-SVF groups (19.6 ± 4.8 vs. 17.2 ± 3.2, respectively, *p* = 0.42; Fig. 8a, b). Additionally, both groups showed formation of vascular tube-like structures and demonstrated high potential for tube formation without significant differences between the F- and C-SVF groups (8.7 ± 3.5 vs. 7.2 ± 2.9 mm, respectively; *p* = 0.31; Fig. 8c, d).

Regarding osteogenic differentiation, monolayer cultures of the F- and C-SVF groups were positive for ALP and alizarin red staining (Fig. 8e). There were no significant differences in ALP activity and alizarin red mineralization between the F- and C-SVF groups (ALP activity: 187.7 ± 22.4 vs. 165.4 ± 22.8 × 10 ng/mL, *p* = 0.28; alizarin red mineralization: 1.80 ± 1.49 vs. 1.70 ± 0.99, *p* = 0.90 Fig. 8f, g).

## Discussion

The main findings of this study were that (1) transplantation of human SVF cells at the site of a nonunion fracture in an immunodeficient rat model could enhance bone healing in terms of radiological, histological, and biomechanical properties, and (2) cryopreserved SVF cells exhibit a therapeutic effect equivalent to that of fresh specimens. To the best of our knowledge, this study is the first to compare the therapeutic potential of fresh vs. frozen specimens for fracture healing. Based on these findings, transplantation of SVF cells is expected to be a feasible treatment option for nonunion in the near future and cryopreservation could be useful to efficiently preserve SVF cells while maintaining angiogenic and osteogenic potentials.

Our results show that local transplantation of F- and C-SVFs promoted radiological ossification more efficiently as compared to the control group, and 50–60% of fractures were healed at POW 8. Additionally, plain radiographs revealed that the density of the callus area was greater in the F- and C-SVF groups than the control group, and micro-CT showed higher bone mineral content and volume fraction of the callus in both SVF groups than the control group at POW 8. This outcome is supported by histological evaluation based on the classification scheme proposed by Allen et al. [36]. Additionally, in the early process of fracture healing at POW 2, immunostaining of both SVF groups demonstrated capillary perfusion via neovascularization and the enhancement of osteogenesis. These favorable observations in the SVF groups led to improved functional recovery of nonunion fractures as confirmed by the biomechanical 3-point bending test. Similarly, Nomura et al. [30] reported promotion of bone formation via angiogenesis and osteogenesis in a rat model of distraction osteogenesis, and Shoji et al. [37] reported that transplantation of ADSCs accelerated bone healing in a rat model of non-healing fracture via enhancement of osteogenesis and angiogenesis. Our results agree with those of these previous studies. The present study is the first to test the therapeutic potential of SVF cells using a rat model of non-healing fractures and strengthens the hypothesis that adipose-derived tissue can promote fracture healing. However, the rate of fracture union using SVF cells in this study was relatively lower than with the use of ADSCs (50–60% vs. 90%, respectively) [37], which could be due to difference in the preparation of the animal model and/or cell composition, especially the number of transplanted ADSCs. Hence, in future studies, comparisons of ADSCs and SVF cells in the same animal model under identical conditions should be considered.

The results of immunofluorescence analysis showed that intrinsic angiogenesis and osteogenesis were enhanced to a greater extent in both SVF groups than the control group. Additionally, RT-PCR analysis with rat-specific primers showed that the expression levels of osteogenic and angiogenic cytokines (BMP2 and VEGF) were upregulated in both SVF groups as compared to the control group. These results indicate that the mechanisms underlying the osteogenic and angiogenic effects of SVF cells might involve paracrine effects on resident cells and that interactions between transplanted human SVFs and resident cells are critical for fracture repair. Previous studies [47–50] have shown that transplantation of adipose tissue can promote tissue regeneration through the secretion of various cytokines, as well as increased expression of angiogenic cytokines (HGF, VEGF), hematopoietic cytokines (G-CSF), and cytokines that promote bone formation (BMP-2). We also observed upregulated expression of HIF1-a in both SVF groups. HIF, which is an upstream molecule of VEGF and the angiopoietin-1 signaling pathway, is reportedly upregulated under hypoxic conditions in vitro [51]. Our results also indicate that the SVF cells produced HIF1-a at the hypoxic fracture site created by cauterizing the periosteum during preparation of the nonunion model and, thereby, enhanced intrinsic angiogenesis through upregulation of VEGF.

Immunohistochemical analysis showed that transplantation of human ECs and OBs at the fracture sites promoted differentiation into osteoblastic and endothelial lineages, which might be a mechanism underlying the osteogenic and angiogenic effects of SVF cells. Based on these findings, we next examined the differentiation potential of F- and C-SVF cells in vitro and found a high potential for the formation of vascular tube-like structures and remarkable osteogenesis potential, as revealed by ALP and alizarin red staining. Several studies of tissue regeneration with the use of ADSCs have provided direct evidence for the differentiation of ADSCs into multiple cell lineages in vitro as well as in vivo [30, 37, 52–54]. ADSCs have been shown to differentiate into ECs secreting VEGF and leptin when transplanted in the ischemic hind-limb of mice [52]. Other studies have reported the osteogenic capability of ADSCs using a mouse model of calvarial defects [53] and the chondrogenic multipotency of ADSCs for regeneration of articular cartilage [54]. The results of the present study are in line with those of previous studies. However, there were significantly fewer human ECs and OBs than resident cells at week 2, as determined via immunofluorescence analysis. These results suggest that the therapeutic effect of SVF cells is mainly dependent on a paracrine effect rather than differentiation potential, probably because SVF contains many kinds of cells and their progenitors despite the low proportions (2–16%) of multipotent stem cells [55, 56]. Thus, a comparative study between SVF cells and ADSCs using the same animal model would be desirable in the future to reveal the detailed mechanism of bone healing.

Regarding cell viability, all tested parameters of fracture healing were similar between models of cryopreserved and freshly isolated SVF cells. Previous studies have demonstrated no significant effect of cryopreservation on the viability, proliferation, and differentiation of ADSCs [57–59], which is consistent with our results. Lee et al. [60] reported that 10% DMSO was sufficient to reduce apoptosis of ADSCs in vitro. In this study, DMSO was used as a freezing medium, which led to favorable results in fracture healing in the cryopreserved group. However, we observed a 10% decrease in bone union rate in the cryopreservation group compared to the fresh SVF group. This suggests that the cryopreservation period could potentially influence cell viability and therapeutic effect. Although a study conducted on the ADSC stored for longer than 10 years has reported that long-term cryopreservation maintains cell viability with certain negative effects on osteogenic potential of ADSCs [61], for better clarity on SVF, comparison between different cryopreservation periods using the same animal model is ideal and should be considered. Several recent studies have reported the therapeutic potential of cryopreserved SVF has for bone healing in equine fracture [33] and rat bone defect models [34]. However, to the best of our knowledge, our study is the first to compare the therapeutic benefits of fracture healing using the same animal unhealing fracture model between freshly isolated and cryopreserved SVFs, and strengthen the evidence of usefulness of cryopreservation of SVFs to efficiently maintain angiogenic and osteogenic potentials.

There were some limitations to this study that should be addressed. First, the number of samples was small, which limits the ability to make generalized conclusions. A larger sample size is ideal to discern differences in the therapeutic effects between the F- and C-SVF groups. Second, only female rats were tested because females are less likely to fight and incur leg injuries. However, male rats could have been used as a nonunion model, thus future studies of both male and female rats should be considered. Third, we used immunodeficient rats as a model since it is commonly used in similar studies on xenotransplantation [9, 37, 41], which prevents grafted human tissue/cell rejection. However, several articles have reported that the specific immune-response signal accelerates bone healing during the late stage of fracture repair [62, 63]. Thus, immunodeficiency condition may interfere with the outcome of practical therapeutic effects of SVF. Therefore, challenges remain in directly translating the results of this study into clinical practice. Fourth, we experienced 3 deaths and 3 infections in 96 operations. Three dead animals were included in the initial 20 cases of the operation and died on the day of surgery or the day following. Therefore, the cause of death was attributed to intraoperative blood loss due to the immaturity of the surgical procedure and the prolonged surgery time. Although the primary cause of infection remains unknown, the prolonged surgery time and immunodeficient conditions might have a significant influence. Based on the above, a treatment by an experienced surgeon is desired in clinical setting and indication for immunodeficient patient should be carefully considered. Fifth, the adipogenic potential of SVFs are mutually exclusive to osteogenic potential in bone healing. In this study, we focused on the osteogenesis and angiogenesis and did not assess the negative effect of the adipogenesis at the fracture site on bone healing, which should therefore be assessed in future studies. Finally, based on our results, about half of the rats did not achieve bone union even after SVF administration. Thus, it is necessary to identify factors influencing bone union in SVF treatment.

## Conclusions

In summary, human SVF cells were transplanted in immunodeficient rats for the treatment of non-healing fractures. SVF cell administration radiologically, histologically, and biomechanically enhanced fracture healing via intrinsic angiogenesis/osteogenesis and human cell-derived vasculogenesis/osteogenesis. These results confirm that SVF treatment can improve fracture repair, suggesting that transplantation of SVF cells is a promising strategy for the treatment of nonunion in future clinical settings. Furthermore, our data established that cryopreserved specimens have almost equal potential for fracture healing as fresh specimens, indicating that the effect of SVF cells on angiogenesis and osteogenesis can be maintained under cryopreservation.

## Data Availability

The datasets generated and/or analyzed during the current study are not publicly available but are available from the corresponding author upon reasonable request.

## Abbreviations

ADSC: Adipose-derived stem cells
ALP: Alkaline phosphatase
CT: Computed tomography
EC: Endothelial cell
FBS: Fetal bovine serum
GA: Gentamicin, amphotericin-B
HE: Hematoxylin and eosin
IFATS: International Federation for Adipose Therapeutics and Science
ISCT: International Society for Cellular Therapy
PBS: Phosphate-buffered saline
SD: Standard deviation
SVF: Stromal vascular fraction
